# The Evolving Landscape of Respiratory Syncytial Virus (RSV) Prophylaxis in Infants

**DOI:** 10.7759/cureus.97521

**Published:** 2025-11-22

**Authors:** Sastry Chamarthi, Surendra Gupta, Venkata Sushma Chamarthi

**Affiliations:** 1 Pediatrics, Clinica Sierra Vista Elm Community Health Center, Fresno, USA; 2 Pediatrics, Valley Children’s Healthcare, Fresno, USA

**Keywords:** infants, maternal immunization, nirsevimab, prophylaxis, rsv

## Abstract

Throughout human history, respiratory infections have been among the most common illnesses affecting populations worldwide. Respiratory syncytial virus (RSV) is recognized as one of the major pathogens responsible for a significant disease burden in young children and the elderly population, causing lower respiratory tract infections. Initially identified as the chimpanzee coryza virus in the mid-20th century, it was later named human RSV. This review outlines the discovery of RSV, the initial challenges faced in vaccine development, and the evolution of monoclonal antibodies for infants and vaccines for pregnant mothers to prevent RSV infection in infants. Early vaccine trials enhanced respiratory disease and increased mortality among vaccinated infants. There were subsequent failures with live attenuated vaccines. Research turned toward developing the monoclonal antibody palivizumab, which provided the first practical means of protecting high-risk infants against RSV infection. Subsequent advances in monoclonal antibodies, especially nirsevimab, and the advent of the maternal vaccination program with the Bivalent Prefusion F Vaccination have expanded disease prevention to the population level instead of targeting high-risk infants. These advances promise to reduce hospitalizations and severe disease, improving overall community health. Future priorities include ensuring equitable access, maintaining long-term safety surveillance, and integrating RSV prevention into national immunization programs worldwide.

## Introduction and background

Respiratory syncytial virus (RSV) was discovered serendipitously in 1955 at the Walter Reed Army Institute of Research. Morris, Blount, and Savage isolated a novel virus from chimpanzees suffering from respiratory illness at the Walter Reed Army Institute of Research. The virus was initially named chimpanzee coryza agent (CCA) or chimpanzee coryza virus (CCV) in 1956 [1]. In 1957, Chanock and colleagues at Johns Hopkins University isolated a virus associated with severe respiratory illness in infants, similar to the one causing illness in Baltimore [2]. When serological studies revealed that most school-aged children possessed neutralizing antibodies to this agent, Chanock proposed renaming it "Respiratory Syncytial Virus" to reflect its human pathogenicity and characteristic induction of syncytia formation in infected cells [2,3]. This virus was subsequently identified as the cause of lower respiratory tract disease in infants and young children, causing bronchiolitis and pneumonia, marking the beginning of RSV's recognition as a major pediatric respiratory pathogen [3].

Further research led to an understanding of viral structure and the mechanism of pathogenesis [4]. RSV is an enveloped single-stranded RNA virus containing non-structural and structural proteins. The two structural glycoproteins are the fusion F protein and the attachment G protein, present on the surface of the viral envelope [4]. These two play a significant role in pathogenicity and infectivity. There are antigenic differences in the protein G; RSV is classified into RSV A and RSV B based on this [5]. An attachment of the G protein to host cells activates the F protein. The fusion of the viral envelope with the host cell triggers a conformational change in the F protein, transitioning it from a prefusion to a postfusion state [4]. This fusion and subsequent spread lead to the characteristic formation of multinucleated cells, or syncytia, which gives the virus its name. This syncytium formation is the basis for the virus's name, respiratory syncytial virus. Due to its critical role in the fusion process, the F protein has been studied extensively for the creation of neutralizing antibodies, and understanding its conformational states is vital for developing modern disease prevention and vaccination strategies [4,5].

Respiratory syncytial virus (RSV) represents a major cause of acute lower respiratory tract infections globally, resulting in substantial morbidity and mortality among infants and young children [6]. Between 2023 and 2025, RSV prevention shifted from risk-targeted to population-level strategies. The World Health Organization's Strategic Advisory Group of Experts on Immunization (SAGE) issued its first global RSV immunization recommendation in September 2024, advising all countries to introduce either maternal RSV prefusion F protein (RSVpreF) vaccination or long-acting monoclonal antibodies based on feasibility and cost-effectiveness. WHO prequalification of maternal RSV vaccine in March 2025 enabled broader access through United Nations (UN) agencies and Gavi, the vaccine alliance, particularly benefiting low- and middle-income countries where 97% of pediatric RSV deaths occur. Cost-effectiveness analyses demonstrated that these interventions could prevent thousands of hospitalizations and deaths annually, while reducing overall healthcare costs, thereby elevating RSV prevention to a global health priority that is integrated into routine immunization and antenatal care programs [7].

## Review

Methods

We conducted this narrative review to synthesize the current evidence on the evolution and implementation of RSV prophylaxis. PubMed searches were initiated in early 2024 and updated through October 2025 using combinations of the following search terms: "respiratory syncytial virus," "RSV," "nirsevimab," "clesrovimab," "palivizumab," "monoclonal antibody," "maternal vaccination," "RSVpreF," "pregnancy," "prophylaxis," "prevention," and "immunization." We supplemented database searches with official public health sources, including guidance from the Centers for Disease Control and Prevention (CDC), Advisory Committee on Immunization Practices (ACIP) recommendations, policy statements from the American Academy of Pediatrics (AAP), and position papers from the World Health Organization (WHO) published through 2025.

We prioritized randomized controlled trials, meta-analyses, large observational studies, real-world effectiveness evaluations, analyses from the New Vaccine Surveillance Network (NVSN), and major policy statements from national and international agencies. Literature was limited to English-language publications. Gray literature from authoritative public health agencies (CDC Morbidity and Mortality Weekly Report (MMWR) reports, WHO position papers, regulatory guidance) was included, given its critical role in policy implementation.

All authors reviewed, summarized, and tabulated the data narratively and chronologically, categorizing the results by study design (randomized controlled trials, cohort studies, case-control studies, meta-analyses, and systematic reviews). Given extensive existing evidence supporting palivizumab in protecting preterm and high-risk infants, this review emphasizes newer monoclonal antibodies (nirsevimab, clesrovimab), recent maternal vaccine trials, contemporary meta-analyses, and systematic reviews published since 2020. As a narrative review, formal risk of bias assessment tools were not applied. However, we prioritized peer-reviewed publications, large multicenter trials, and official guidance from recognized public health authorities to ensure the quality and reliability of the evidence.

Current epidemiological landscape and vulnerable populations

RSV remains a leading cause of acute lower respiratory infection among young children worldwide, with approximately 33 million episodes and over 100,000 deaths annually in children under five years of age, predominantly in low- and middle-income countries [6]. In the United States, RSV is the leading cause of infant hospitalization, with 58,000-80,000 annual pediatric admissions [8,9]. Risk is substantially elevated in premature infants, those with congenital heart or chronic lung disease, and immunocompromised children and is influenced by socioeconomic factors, including poverty, environmental exposures, and race/ethnicity [6,8-10]. Introduction of maternal vaccination and long-acting monoclonal antibodies has begun to decrease the burden of RSV disease [7]. Detailed epidemiological data, risk factors, and high-risk populations are summarized in Table 1.

**Table 1:** Global and U.S. Burden of RSV and High-Risk Populations RSV: respiratory syncytial virus, LRTI: lower respiratory tract infection, CHD: congenital heart disease, CLD: chronic lung disease, BPD: bronchopulmonary dysplasia. The table was created by author Sastry Chamarthi based on the references in the table.

Parameter	Estimates/Key Findings
RSV-LRTI worldwide (children <5 yrs) [6]	~33 million annually
RSV hospitalizations worldwide (children <5 yrs) [6]	~3.6 million annually
RSV-related primary care visits (North America and Europe) [11]	0.8 to 330 (median = 109) per 1,000 population
RSV-related emergency department visits [11]	7.5 to 144 (median = 48)
RSV-related deaths worldwide (children <5 yrs) [6]	~101,000 deaths annually; 97% in low- and middle-income countries
RSV hospitalizations (United States) [8,9]	58,000-80,000 hospital admissions annually under 5 yrs of age, with infants less than 1 yr being primary proportion
Highest-risk age group [8]	0-6 months, especially preterm and those with CHD or CLD
Common comorbidities [8]	Prematurity, CHD, CLD, BPD, immunodeficiency
Socioeconomic/race/ethnicity/environmental factors [6,8,9]	Risk is higher with poverty, smoke exposure, and childcare attendance; hospitalization rates are disproportionately higher among non-Hispanic Black, Native American/Alaska Native, and Hispanic infants

Early vaccine lessons and historical milestones

Early efforts to prevent RSV infection began in the 1960s with the development of a formalin-inactivated RSV (FI-RSV) vaccine. FI-RSV vaccination failed to provide protection and instead led to enhanced respiratory disease (ERD) in vaccinated infants, resulting in hospitalization and deaths. Current understanding suggests that FI-RSV vaccine failure resulted from improper antigen processing during formalin inactivation, which disrupted critical conformational epitopes and led to production of non-neutralizing antibodies and aberrant T-cell responses. This generated immune complexes and complement activation upon natural RSV infection, resulting in excessive inflammatory responses and enhanced disease severity [12-14]. Subsequent research into subunit and live-attenuated vaccine platforms encountered safety and long-lasting immunogenicity challenges [12-14].

The antiviral ribavirin was licensed to treat RSV infection in the 1980s. Aerosolized ribavirin was approved for severe RSV infection, but its use declined due to difficulty in administration, poor efficacy, prolonged hospitalizations, and potential teratogenic concerns. While oral inhalation ribavirin remains available and is occasionally used in immunocompromised patients with severe RSV, it is not routinely recommended for otherwise healthy infants [14-16]. The ERD with the FI-RSV vaccine and the limited effectiveness of therapeutics were setbacks that reshaped the understanding of RSV immunopathology, leading to the development of passive immunization strategies.

**Figure 1:**
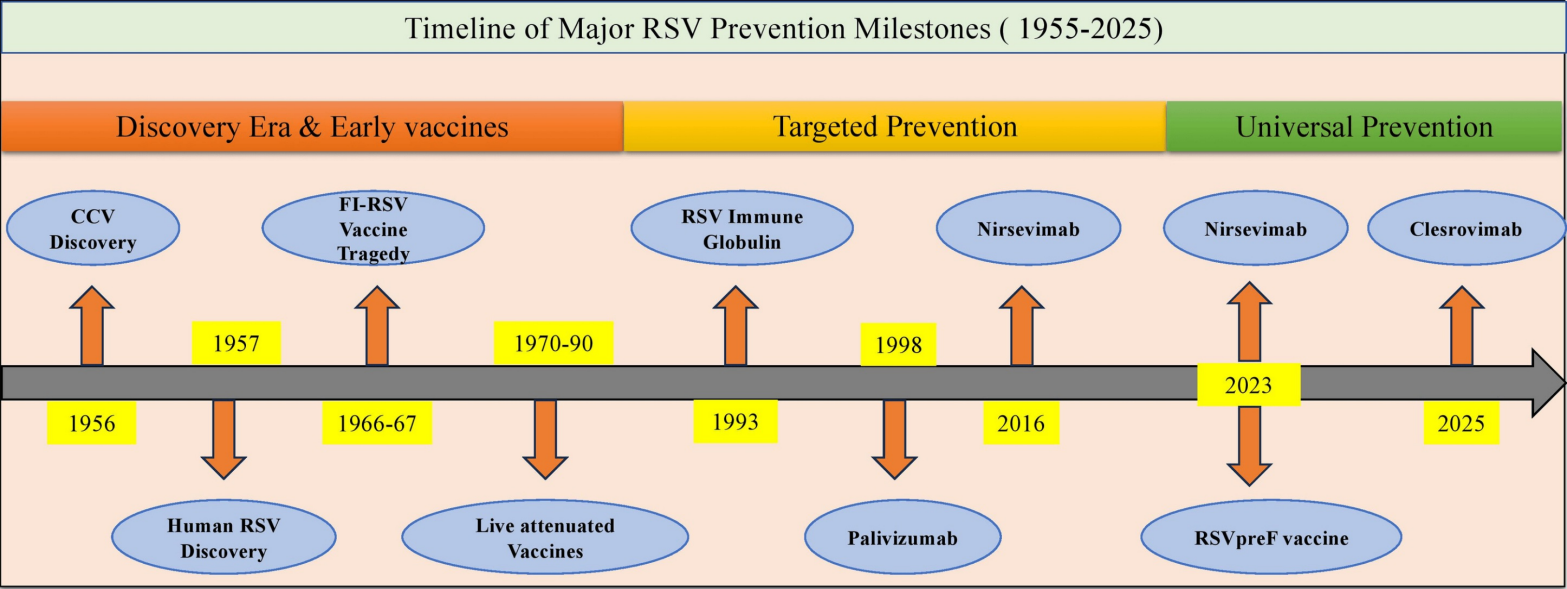
Timeline of Major RSV Prevention Milestones (1955-2025) This timeline summarizes key milestones in the development of respiratory syncytial virus (RSV) prevention strategies over seven decades. The Discovery Era and Early Vaccines (1955-1990) includes the identification of the chimpanzee coryza virus (CCV) and RSV, followed by the formalin-inactivated (FI-RSV) vaccine tragedy and early live-attenuated vaccine trials. The Targeted Prevention Era (1993-2016) highlights the introduction of RSV immune globulin and monoclonal antibodies, including palivizumab and the development of nirsevimab. The Universal Prevention Era (2023-2025) marks the approval of nirsevimab for infant protection, the bivalent RSV prefusion F protein (RSVpreF) maternal vaccine, and emerging candidates such as clesrovimab, reflecting a shift toward population-wide RSV immunization strategies. Image created by authors Sastry Chamarthi and Venkata Sushma Chamarthi.

Success with RSV immune globulin in high-risk infant populations laid the groundwork for developing monoclonal antibody prophylaxis in targeted high-risk infants. Subsequent advances in immunology enable broader protection strategies, leading to universal protection of all infants via maternal immunizations or novel monoclonal antibodies. These advancements marked a decisive shift from targeted prophylaxis to proactive disease prevention in all young children. Figure 1 provides an overview of significant milestones in RSV prevention from 1955 to 2025, depicting the transition from early vaccine trials to targeted monoclonal antibody and universal vaccine approaches (Figure 1).

Passive immunization: targeted protection

Early vaccine trials were associated with setbacks, and research shifted toward passive immunization strategies, utilizing immune globulin and monoclonal antibodies. Initially, RSV immune globulin (RespiGam) targeted broader antigenic sites, but was eventually replaced by monoclonal antibodies with greater specificity.

RSV fusion F glycoprotein facilitates viral entry into the host cell, becoming an antigenic target for the development of monoclonal antibodies. There are six antigenic sites -Ø, I, II, III, IV, and V. Sites Ø and V are exclusive to the prefusion (preF) conformation, while sites II, III, and IV are shared between preF and postfusion (postF) states, with site III exhibiting higher affinity in the preF state. Site I is specific to the postF conformation (Table 2). These insights led to the development of monoclonal antibodies with enhanced neutralization capacity and longer half-lives. Several agents have been approved and discontinued based on clinical trial data [14,17-24].

**Table 2:** Neutralizing Antibodies and Their Antigenic Targets on the RSV Fusion (F) Protein RSV: respiratory syncytial virus. Table created by author Sastry Chamarthi based on references [14,17-24]. EMA: European Medicines Agency.

Antibodies	Target site	Status
RSV immune globulin (RespiGam)	Polyclonal	Discontinued
Palivizumab	Site II (preF and postF)	FDA and EMA approved; to be discontinued by manufacturer in 2025
Nirsevimab	Site Ø (preF)	FDA and EMA approved; currently in clinical use as of 2025
Motavizumab	Site II (preF and postF)	Discontinued at trials in view of adverse effects
Clesrovimab	Site IV	FDA approved; currently in clinical use as of 2025
Suptavumab	Site V (preF)	Failed in reducing hospitalizations; not approved

Maternal active immunization: infant protection

Continued advances in RSV research led to the development of a bivalent prefusion F (preF) protein vaccine. This bivalent vaccine contains stabilized preF antigens derived from both RSV A and RSV B subgroups, ensuring broad protection. The success of this maternal vaccination enables passive transplacental transfer of antibodies to the fetus and protects the infant from RSV beginning from birth [25].

Discussion

Early Passive Immunization: RSV Immune Globulin and Palivizumab

Initial passive immune efforts used RSV immune globulin (RespiGam), administered as monthly intravenous infusions to high-risk infants (congenital heart disease (CHD), bronchopulmonary dysplasia (BPD), or prematurity), were successful in providing 60% protection against RSV and reducing hospitalization days, according to a 1993 study [26]. Success with polyclonal antibody protection led to the development of monoclonal antibody prophylaxis directed at the RSV fusion (F) glycoprotein. The monthly intramuscular palivizumab injections over five months in the multicenter clinical trial IMpact-RSV study showed a significant 55% reduction in RSV-related hospitalizations. It has shown to be promising as a 78% reduction in RSV-related hospitalization among preterm infants less than 35 weeks of gestation without BPD, and a 39% reduction in children with BPD. Subsequent randomized controlled trials performed on children with CHD showed that monthly palivizumab prophylaxis 45% relative reduction in hospitalizations and decreased the length of stay at hospitals. A 2025 Cochrane review evaluating Palivizumab for preventing severe RSV infection confirms that monthly intramuscular injections significantly reduce RSV infections and decrease hospitalizations without a significant effect on mortality [27]. A second-generation monoclonal antibody, motavizumab, which binds to similar antigenic sites as palivizumab, has shown promise by significantly reducing intensive care admissions. However, it was associated with significant adverse skin reactions, leading to the discontinuation of further studies [20,28,29].

Evolution of Long-Acting Monoclonal Antibodies

Despite these benefits, targeted prophylaxis has limitations. Eligibility criteria restrict protection to a small subset of infants, and the need for repeated and seasonal dosing poses a logistical challenge to disease prevention. Research continued for long-acting monoclonal antibodies that could be administered as a single dose for the RSV season. Clinical trials evaluated newer monoclonal antibodies nirsevimab, clesrovimab, and suptavumab. Among them, nirsevimab and clesrovimab were successful in clinical trials, while suptavumab did not decrease RSV hospitalization [22,23]. Single-dose administration has simplified the execution plan to universally protect all infants, instead of targeting high-risk populations.

Efficacy and Safety of Nirsevimab and Clesrovimab

Recent clinical trials, randomized controlled studies, meta-analyses, and systematic reviews confirm that nirsevimab significantly reduces RSV disease and hospitalization, offering the added advantage of a single dose for ease of administration. Similarly, clesrovimab also proved to be effective based on the randomized controlled trial, reducing hospitalization by 84% and RSV disease prevention by 60%. However, suptavumab did not decrease hospitalization, so it was discontinued [23].

Nirsevimab and clesrovimab have similar safety profiles. Commonly reported adverse reactions included transient fever and injection site reactions like erythema. The severe adverse reactions appear to be comparable to placebo in the clinical trials [30,31]. It is also noted a potential increase in co-infection with other respiratory viruses in the NIRSEGRAND study conducted in Spain, in infants who received nirsevimab. This needs further studies to confirm or refute [32]. Additionally, studies have shown that the intranasal route for vaccine and monoclonal antibody delivery was ineffective and associated with adverse reactions [27,33].

Maternal RSV Vaccination: Clinical Trials and Real-World Evidence

The development of a bivalent prefusion F protein (RSVpreF) vaccine for maternal vaccination was studied in the Maternal Immunization Study for Safety and Efficacy (MATISSE) trial, which demonstrated 80% efficacy in preventing RSV disease in the first three months [25]. This was followed by two multicenter real-world studies that confirmed effectiveness, reporting reductions in RSV-associated hospitalizations of 71%-79% in infants up to six months of age [34,35]. A summary of landmark clinical trials and real-world studies assessing RSV immune globulin and monoclonal antibody prophylaxis, including RespiGam, palivizumab, nirsevimab, and clesrovimab, is presented in Table 3. A summary of pivotal maternal RSV vaccine trials and real-world evaluations is presented in Table 4.

**Table 3:** Major Clinical Studies Evaluating Monoclonal Antibody and Immunoglobulin Therapies for RSV Prevention (1993-2025) This table summarizes pivotal studies evaluating respiratory syncytial virus (RSV) immunoglobulin and monoclonal antibody prophylaxis from 1993 to 2025. The data demonstrate progressive advances from polyclonal RSV immune globulin (RespiGam) to targeted monoclonal antibodies such as palivizumab, nirsevimab, and clesrovimab, highlighting their efficacy in reducing RSV-associated lower respiratory tract infections (LRTIs) and hospitalizations across high-risk and general infant populations. RSV: respiratory syncytial virus; LRTI: lower respiratory tract infection; BPD: bronchopulmonary dysplasia; CHD: congenital heart disease; CLD: chronic lung disease; GA: gestational age; IM: intramuscular; OR: odds ratio. Table created by author Sastry Chamarthi based on references cited in the table.

Study Type/Year	Study Title	Study Population	Intervention	Key Outcomes
Clinical Trial (1993) [26]	Prophylactic Administration of Respiratory Syncytial Virus Immune Globulin to High-Risk Infants and Young Children	249 infants (mean age: 8 months) with BPD, CHD, or prematurity	Monthly RSV immune globulin (high-dose, low-dose, or placebo)	High-dose group had fewer LRTIs (7 vs 20; p=0.01) and hospitalizations (6 vs 18; p=0.02)
Multicenter Clinical Trial (1998) [36]	Palivizumab, A Humanized Respiratory Syncytial Virus Monoclonal Antibody, Reduces Hospitalization From Respiratory Syncytial Virus Infection in High-Risk Infants	1,502 children ≤35 weeks GA or with BPD	5 monthly IM injections of palivizumab (15 mg/kg) vs placebo	55% reduction in RSV hospitalization; 78% reduction in preterm infants without BPD; 39% in infants with BPD
Randomized Double-Blind Placebo-Controlled Trial (2003) [37]	Palivizumab Prophylaxis Reduces Hospitalization Due to Respiratory Syncytial Virus in Young Children With Hemodynamically Significant Congenital Heart Disease	1,287 children with CHD	5 monthly IM injections of palivizumab (15 mg/kg) vs placebo	45% reduction in RSV hospitalization (p=0.003); 56% reduction in total hospital days per 100 children (p=0.003)
Randomized Controlled Trial (2020) [38]	Single-Dose Nirsevimab for Prevention of RSV in Preterm Infants	1,453 healthy preterm infants (29-34 + 6 weeks GA)	Single IM injection of nirsevimab (50 mg) vs placebo	RSV-LRTI reduced by 70% (p<0.001); RSV hospitalization reduced by 78% (p<0.001)
Multicenter Clinical Trial (2022) [39]	Safety of Nirsevimab for RSV in Infants With Heart or Lung Disease or Prematurity	900 palivizumab-eligible infants (≤35 weeks GA, CLD, or CHD)	Single IM nirsevimab (50 mg < 5 kg; 100 mg ≥ 5 kg) vs 5 monthly palivizumab injections	RSV-LRTI: 0.6% vs 1.0%; similar safety and immunogenicity
Multicenter Randomized Clinical Trial (2023) [30]	Nirsevimab for Prevention of Hospitalizations Due to RSV in Infants	8,058 infants ≤ 12 months (≥29 weeks GA) in France, Germany, UK	Single IM nirsevimab vs standard care	RSV hospitalization reduced 83%
Retrospective Observational Cohort (2025, NIRSEGRAND, Spain) [32]	Nirsevimab Prophylaxis for Reduction of Respiratory Syncytial Virus Complications in Hospitalised Infants: The Multi-Centre Study During the 2023-2024 Season in Andalusia, Spain (NIRSEGRAND)	222 infants < 6 months in Andalusia (2023-2024)	Nirsevimab vs no prophylaxis	Reduced nasal cannula 64%, ventilation 48%, ICU 54%, hospitalization duration 30%
Systematic Review and Meta-Analysis (2024) [40]	Impact of Nirsevimab Immunization on Pediatric Hospitalization Rates: A Systematic Review and Meta-Analysis (2024)	45,238 infants across 19 studies	Nirsevimab immunization	RSV hospitalization prevention efficacy 88%
Randomized Clinical Trial (2025) [31]	Clesrovimab for Prevention of RSV Disease in Healthy Infants	3,614 healthy preterm and full-term infants entering first RSV season	Single IM 105 mg clesrovimab vs placebo	RSV-LRTI efficacy 60%; hospitalization efficacy 84%
Multicenter Retrospective Cohort (2025, Italy) [41]	Sustained Clinical and Epidemiological Impact of Respiratory Syncytial Virus (RSV) in Young Infants Exposed to Universal Immunization With Nirsevimab at Birth: An Italian Multicenter, Retrospective, Cohort Study 2024/25	309 hospitalizations (140 RSV+, 169 RSV-)	Universal nirsevimab immunization at birth	66% efficacy against RSV-related hospitalization
Multicenter Matched Case-Control (2025, Italy) [42]	Effectiveness of a Targeted Infant RSV Immunization Strategy (2024-2025): A Multicenter Matched Case-Control Study in a High-Surveillance Setting	138 infants (46 cases, 92 controls)	Targeted RSV immunization (nirsevimab)	Immunization efficacy 89%
Meta-Analysis (2025) [43]	Effectiveness of Nirsevimab Immunization Against RSV Infection in Preterm Infants: A Systematic Review and Meta-Analysis	7,347 preterm infants (5 studies)	Nirsevimab immunization	Reduced medically attended RSV-LRTI (OR: 0.25); RSV hospitalization (OR: 0.27)

**Table 4:** Maternal RSV Prefusion F Protein (RSVpreF) Vaccine Trials and Real-World Effectiveness Studies (2023-2025) This table summarizes pivotal maternal RSV prefusion F protein (RSVpreF) vaccine trials and real-world evaluations conducted between 2023 and 2025. Collectively, these studies demonstrate strong protection against RSV-associated lower respiratory tract disease (LRTD) and hospitalization in early infancy, reinforcing the value of maternal immunization as a population-wide prevention strategy. RSV: respiratory syncytial virus; LRTD: lower respiratory tract disease; GA: gestational age; RSVpreF: respiratory syncytial virus prefusion F protein vaccine. Table created by author Sastry Chamarthi based on references cited in the table.

Study Design/Year	Study Title	Study Population	Intervention	Key Outcomes
Phase 3 Randomized, Double-Blind, Placebo-Controlled Trial (2025) [25]	Efficacy, Safety, and Immunogenicity of the MATISSE (Maternal Immunization Study for Safety and Efficacy) Maternal Respiratory Syncytial Virus Prefusion F Protein Vaccine Trial	7,420 pregnant participants	Single maternal intramuscular RSVpreF vaccine vs placebo	Vaccine efficacy: 82% (95% CI, 57.5-93.9) against RSV infection at 90 days; 70% (95% CI, 50.6-82.5) at 180 days post-birth
Multicenter Retrospective Test-Negative Case-Control Study (2025, Argentina) [34]	Real-World Effectiveness of RSVpreF Vaccination During Pregnancy Against RSV-Associated Lower Respiratory Tract Disease Leading to Hospitalisation in Infants During the 2024 RSV Season in Argentina (BERNI Study): A Multicentre, Retrospective, Test-Negative, Case-Control Study	633 infants hospitalized in 12 Argentinian hospitals during 2024 RSV season	Maternal RSVpreF vaccination at 32 + 0 to 36 + 6 weeks GA, ≥14 days before delivery	Effectiveness: 78.6% (95% CI, 62.1-87.9) against RSV-LRTD hospitalization (0-3 months); 71.3% (95% CI, 53.3-82.3) from 0 to 6 months; 76.9% for severe LRTD (0-6 months)
Multicenter Test-Negative Case-Control Study (2025, UK) [35]	Bivalent Prefusion F Vaccination in Pregnancy and Respiratory Syncytial Virus Hospitalisation in Infants in the UK: Results of a Multicentre, Test-Negative, Case-Control Study	537 mother-infant pairs	Maternal RSVpreF vaccination any time before delivery	Effectiveness: 58% (95% CI, 28-75) overall; 72% if >14 days before delivery

Guideline evolution

With advances in active immunization and the availability of longer-acting monoclonal antibodies for infants, RSV prevention guidelines have evolved and adapted to incorporate modifications based on ongoing research. For 2025-2026, RSV season, Centers for Disease Control and Prevention (CDC), the American College of Obstetricians and Gynecologists (ACOG), and the Advisory Committee on Immunization Practices (ACIP) immunization during pregnancy for prevention of RSV infection in infants include maternal RSV vaccination between 32 and 36 weeks of gestation from September through January in the United States [44]. This timing optimizes the transfer of placental antibodies against the preF protein while minimizing any potential risk of preterm birth. Optimal antibody transfer typically occurs approximately two weeks before delivery. Guidance also suggests that if the mother received the RSV vaccine in a previous pregnancy, revaccination is not needed in the current pregnancy. The CDC is continuing to monitor data on whether revaccinations may be required in future pregnancies.

**Figure 2:**
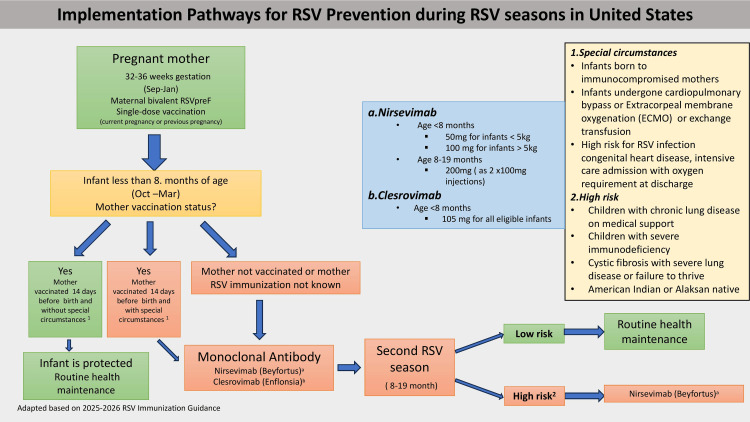
Implementation Pathways for RSV Prevention During RSV Seasons in the United States This figure illustrates the recommended decision pathways for respiratory syncytial virus (RSV) prevention in infants and pregnant mothers based on current Centers for Disease Control and Prevention (CDC) guidance. Maternal vaccination with the bivalent RSV prefusion F protein (RSVpreF) vaccine between 32 and 36 weeks gestation (September-January) provides passive protection to infants less than eight months old during the RSV season (October-March). For infants whose mothers were unvaccinated, vaccinated within 14 days of delivery, or whose vaccination status is unknown, monoclonal antibody prophylaxis (nirsevimab or clesrovimab) is indicated. Additional considerations are given to special circumstances (e.g., infants born to immunocompromised mothers or with congenital heart disease) and high-risk groups (e.g., children with chronic lung disease, severe immunodeficiency, or certain ethnic backgrounds). Infants entering a second RSV season (8-19 months) receive prophylaxis if classified as high-risk. The flow diagram emphasizes integration of maternal immunization and monoclonal antibody use for comprehensive RSV prevention. Figure created by authors Sastry Chamarthi and Venkata Sushma Chamarthi, adapted from 2025-2026 CDC RSV Immunization Guidance [44,45].

Postnatally, the current clinical guidance from the CDC, AAP, and ACIP for the 2025-2026 RSV season advises monoclonal antibody prophylaxis in specific situations: if the mother was unvaccinated in pregnancy, or has an immune deficiency disorder, or the infant underwent cardiopulmonary bypass or exchange transfusion leading to depletion of maternal antibodies. The two approved monoclonal antibodies are nirsevimab or clesrovimab, to be administered shortly after birth (October to March) or at the start of the RSV season. Palivizumab is scheduled to be discontinued at the end of 2025. Additional prophylaxis is recommended for children entering a second season, infants with chronic lung disease, congenital heart disease, severe immunocompromise, or specific American Indian/Alaska Native populations [45]. The recommended timing of administration and target groups is summarized in Figure 2. We highlighted policy recommendations and current clinical guidance from CDC, AAP, and ACIP from the United States for the 2025-2026 RSV season.

Implementation realities

In the absence of effective antiviral therapy for RSV, disease prevention is the cornerstone of mitigating RSV burden. Maternal and infant immunization significantly reduces RSV infection and hospitalization, leading to fewer lost workdays for caregivers and a positive economic impact [7]. Successful implementation requires empowering healthcare providers through continuing medical education, webinars, and updated clinical guidelines. Education should extend to nurses, pharmacists, and allied healthcare professionals to facilitate patient counseling and referrals. Hospital systems should integrate RSV immunization protocols into electronic health records to capture maternal vaccination status and identify gaps in coverage. RSV immunization should be offered in conjunction with routine vaccinations during prenatal visits, at pediatric offices, and in hospitals. Administration of RSV monoclonal antibodies at birth for eligible infants during the RSV season should become standard newborn care.

Future directions

Ensuring equitable global access to RSV prevention strategies requires international collaboration to reduce costs and improve accessibility, particularly for low- and middle-income countries. Updated epidemiological studies are needed to reflect the current post-implementation landscape. Further research should evaluate safety outcomes in infants who may inadvertently receive both maternal and infant immunizations and assess whether multifaceted immunization strategies targeting pregnant women, infants, and older adults could contribute to herd immunity and broader community protection.

As RSV prevention measures expand, ongoing surveillance is essential to monitor changes in respiratory virus epidemiology and circulation patterns. Given that RSV infection predisposes individuals to secondary bacterial infections [46-48], future investigations should determine whether widespread RSV prevention reduces the rates of secondary bacterial infections and subsequent antibiotic use.

Limitations

This work is a narrative review, and therefore, it is subject to selection and publication bias. We did not perform a protocolized search or meta-analysis, and study heterogeneity (in terms of populations, case definitions, dosing, and endpoints) limits cross-trial comparability. Our emphasis was on recent infant-focused prevention strategies (long-acting monoclonal antibodies and maternal RSVpreF vaccination) and current U.S. guidance. The country-specific policies, WHO recommendations, and implementation models in low- and middle-income countries were not comprehensively reviewed. We did not examine live-attenuated intranasal vaccine [49] candidates in detail, adult immunization programs, or downstream outcomes such as recurrent wheeze/atopy and asthma phenotypes. Much of the post-marketing evidence for nirsevimab and clesrovimab is limited to early and observational studies. Finally, guidance cited reflects the 2025-2026 season and may evolve with emerging data on revaccination in subsequent pregnancies, supply dynamics, and cost-effectiveness across diverse health systems.

## Conclusions

RSV remains a leading cause of severe lower respiratory tract disease in early infancy. The convergence of maternal immunization with the bivalent RSVpreF vaccine and single-dose, long-acting monoclonal antibodies for infants (e.g., nirsevimab, clesrovimab) represents a step-change from risk-targeted prophylaxis to population-level prevention. Across randomized trials and early real-world studies, these tools consistently reduce medically attended RSV-LRTI and hospitalizations, with safety profiles comparable to placebo in trials and manageable local/systemic reactions in practice.

Clinical pathways now integrate timing of maternal vaccination (32-36 weeks’ gestation) with infant monoclonal antibody use for those with inadequate maternal antibody transfer or ongoing high risk, aligning with current U.S. recommendations. Given that these are newly introduced immunizations, ongoing surveillance is essential for monitoring safety, effectiveness, and long-term impact. Because RSV is a global threat to child health, expanding access to these immunizations in low- and middle-income countries is imperative. Achieving equitable protection will require coordinated investment in research, implementation science, and health policy, alongside financing and supply chain solutions, to translate scientific advances into measurable, population-level gains in child health.
